# Oil for health in sub-Saharan Africa: health systems in a 'resource curse' environment

**DOI:** 10.1186/1744-8603-4-10

**Published:** 2008-10-21

**Authors:** Philippe Calain

**Affiliations:** 1 21 Pont Castelain, 6500 Beaumont, Belgium

## Abstract

**Background:**

In a restricted sense, the resource curse is a theory that explains the inverse relationship classically seen between dependence on natural resources and economic growth. It defines a peculiar economic and political environment, epitomised by oil extraction in sub-Saharan Africa.

**Methods:**

Based on secondary research and illustrations from four oil-rich geographical areas (the Niger Delta region of Nigeria, Angola, southern Chad, Southern Sudan), I propose a framework for analysing the effects of the resource curse on the structure of health systems at sub-national levels. Qualitative attributes are emphasised. The role of the corporate sector, the influence of conflicts, and the value of classical mitigation measures (such as health impact assessments) are further examined.

**Results:**

Health systems in a resource curse environment are classically fractured into tripartite components, including governmental health agencies, non-profit non-governmental organisations, and the corporate extractive sector. The three components entertain a range of contractual relationships generally based on operational considerations which are withdrawn from social or community values. Characterisation of agencies in this system should also include: values, operating principles, legitimacy and operational spaces. From this approach, it appears that community health is at the same time marginalised and instrumentalised toward economic and corporate interests in resource curse settings.

**Conclusion:**

From a public health point of view, the resource curse represents a fundamental failure of dominant development theories, rather than a delay in creating the proper economy and governance environment for social progress. The scope of research on the resource curse should be broadened to include more accurate or comprehensive indicators of destitution (including health components) and more open perspectives on causal mechanisms.

## Background

The soils of most of African countries are rich in mineral, oil or gas resources [1], and could allegedly be exploited for the benefit of resident populations, through domestic processing, exports to world or regional markets, or foreign direct investments (FDI). Mainstream development theories imply that such wealth should have brought about improved livelihoods and better quality of life in sub-Saharan Africa (SSA), after more than four decades past since independence of the continent was officially proclaimed. Accordingly, international financial institutions entertain a carefully optimistic discourse about very recent signs of economic growth in the region [2]. Yet, social indicators of development have shown utterly slow progress over the last one or two decades, as SSA is clearly lagging behind other parts of the world [3]. Marginalised in their pursuit of traditional lifestyles, settled at the insecure margins of fast expanding urban landscapes, or driven in an apparently inescapable transition between both conditions, large segments of sub-Saharan populations are still living in extreme poverty whilst stepping on untapped wealth. This 'paradox of plenty' is common in the developing world, but some of its most striking expressions can be found on the African continent. From a macro-economic perspective, a linked phenomenon can be observed recurrently among oil or mineral producing countries. Following landmark research by Sachs and Warner [4], economists now use the common qualifiers of 'curse of natural resources', 'resource curse' or 'oil curse' [5] to encapsulate the core finding that countries with great natural resource wealth tend to achieve economic growth more slowly than resource-poor countries. Supporting econometric correlations are robust, they are not confounded by geographical or climate variables [6], and they are reproducible [7]. The type of resources that depress economic growth, the so-called 'point-source natural resources', are those whose rents are technically easy to appropriate, such as oil, gas, diamonds, gold, and other minerals [8]. Another feature of such resources is that they are capital intensive in their extraction process and do not generate much employment opportunities [9]. A common assumption is that dependence on the *export *of these commodities is the primary explanatory variable to the resource curse. Considering the extreme and deepening subjection of industrialised nations toward fossil energy, this economic approach to the resource curse would thus explain- as a first approximation – why the case is today nowhere better illustrated than in sub-Saharan Africa, the fastest growing oil-producing region worldwide [10]. As a concept, the resource curse has attracted increasing interest during recent years, among both academic fora and development organisations. Additional findings to the original econometric observations by Sachs and Warner have brought about important considerations that show the intrinsic complexity of the phenomenon. My categorisation of resource curse findings draws mostly upon introductory paragraphs found in papers by Pegg [9,11], Ross [12], Karl [13], and Humphreys *et al*. [14] (Chapter 1). First, a number of *economic mechanisms *have been examined as possible explanatory arguments. These explanations classically encompass processes such as: (i) the loss of economic diversification, following the appreciation of the domestic exchange rate caused by exports of natural resources (the 'Dutch disease'), and (ii) the volatility of the price of fossil fuels. Second, resource curse countries are characterised by high *corruption *levels. The theoretical framework behind this observation relies on the concept of 'rentier state', whereby governments in a capacity to rule in the absence of a functioning tax system are less accountable for misallocation of resources and poor governance. Third, states that rely heavily on oil exports are more likely to adopt *authoritarian *modes of governance. Fourth, the presence of natural resources increases the risk of *civil wars*. While this set of findings focusing on politico-economic mechanisms provides essential pieces to the overall picture describing resource curse environments, causal mechanisms and exact interactions are incompletely understood. The initial econometric definition of the 'curse of natural resources' is a useful framework to approach the counter-intuitive observation that economic growth is hampered by the availability of domestic mineral resources. However, this angle of analysis is clearly reductionist, for at least two reasons. First, much of the macro-economic framework (comparing growth performance between countries) is oblivious of sub-national or local differences *within *countries, and conceals deeper adverse effects for the very populations residing in mineral rich areas. Second, the focus on economic growth to describe the nature of the resource curse assumes that other dimensions (social, political, cultural) are subsidiary to economic factors. Fortunately, some scholars have examined the effect of extractive industries from a broader perspective and used more comprehensive indicators of deprivation than purely economic ones. For example, Gylfason [7] has shown inverse correlations between natural resource abundance and indicators of education level. Using country-wide datasets Ross [15] has observed that 'oil and mineral dependent states tend to suffer from exceptionally high rates of child mortality and low life expectancy' ^a ^and that 'oil dependence is also associated with high rates of child malnutrition; low spending levels on health care; low enrolment rates in primary and secondary schools; and low rates of adult literacy'. More recently, Ross [14] (Chapter 9) has explored the effects of mineral wealth on inequality, pointing out the paucity of available data on vertical income inequalities, i.e. inequalities between social classes. Therefore, it is possible to transcend the reductionist bias carried over by a mere macro-economic perspective on the resource curse and, beyond governance mechanisms, to examine instead the social geography of extractive areas through the lens of proximate determinants of the quality of life, health in particular being an essential one. There are conflicting views about the ultimate benefits or damages to public health, resulting from the exploitation of mineral resources. Adverse health outcomes and impacts are often mentioned in the academic literature addressing the resource curse, but they are generally analysed as peripheral consequences of sustained poverty, insecurity or environmental degradation. On the other hand, industrialists and other proponents of the extensive exploitation of mineral resources tend to justify their position by alleging long-term benefits for health care infrastructures, through economic spillovers of extractive activities. Due to a lack of reliable census data and of health indicators measured over long time periods, it is generally impossible to provide direct quantifications of the net health effects sustained at sub-national level, within the territorial boundaries where extractive industries operate. However, some qualitative elements pertaining to health care delivery in resource curse environments can be analysed in a systematic way. For example in the case of onshore oil-producing areas, the presence of extractive industries can introduce profound societal changes (e.g. forced or voluntary relocation of indigenous populations, human rights abuses, conflicts, urbanisation) that impact on access to health care and on the build up of health systems. The purpose of this article is precisely to contribute to a qualitative description of the public health dimension of the resource curse, taking oil extraction in sub-Saharan Africa as case in point. Considering the broader links that health systems entertain with economic, political and social contexts, resource curse theories are a necessary entrance gate for health system research in mineral-rich areas impacted by extractive industries.

## Methods

The scientific literature (and biomedical sources in particular) does not provide so far any comprehensive description of health systems in specific resource curse environments. As a first approach to fill this gap, I carried out secondary (desk) research to identify existing data about health outcomes/impacts and about components of health systems in oil-producing countries located in sub-Saharan Africa. These countries are listed in reference [10]. I extended country-specific explorations through web-based generic search engines, using the snowball method to retrieve significant references. I focused the search on papers by academic, development or non-profit organisations. Most of the information relevant to health systems is fragmented but converges toward four oil-rich areas: the Niger Delta region of Nigeria, Angola, southern Chad and Southern Sudan. Accordingly, these four settings were selected for illustrative examples. Alongside a selection of classical development indicators, Additional file 1 summarises data to illustrate the variety of contexts among the four selected settings, in terms of history, ongoing conflicts, and importance of oil exploitation. More elaborate narrative summaries of contexts are provided in Appendix 1. Additional file 1 also includes the case of Norway as a benchmark and for reasons considered in the discussion section.

**Figure 1:**
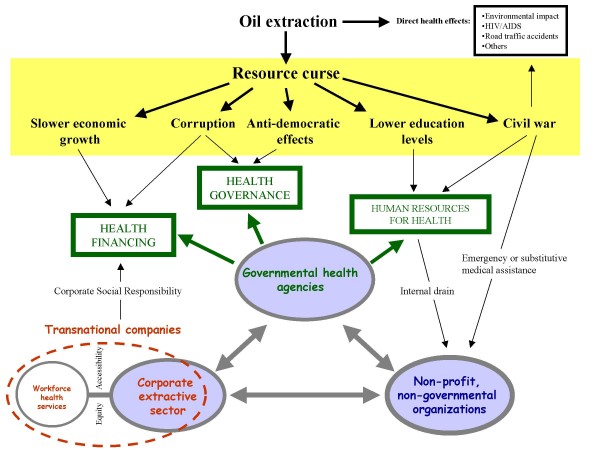
**An analytical framework for health systems in resource curse environments**. The lower two thirds of the figure illustrate the proposed framework for health systems analysis, while elements indicated in the yellow box summarise current findings that characterise resource curse environments. The three core categories of official providers of health services open to local populations are depicted by large shaded grey circles. The realm of transnational oil companies is indicated in red features. Grey double arrows show reciprocal partnerships or contractual relations (see details in the corresponding section of the main text). The main functions classically falling under the responsibility of governmental health agencies are represented by green boxes. Unless specified by captions, plausible influences indicated by thin black arrows represent adverse effects.

Based on this compilation of country data and on a review of the resource curse literature, I first propose a possible generic analytical framework (Figure 1) to define health services available in resource curse environments, including relationships by which they interact, and plausible links with resource curse findings summarised in the previous section. Beside the typology of agencies involved in health services delivery, additional elements to the framework emphasise cultural and institutional values that underpin their activities, operating modes, and respective spaces of legitimacy in which they operate (Table 1). The analysis also addresses the nature of contractual relationships between these categories of agencies, in an attempt to see how much they can contribute to the build-up of a coherent and equitable health system. Prospects for access to health services by indigenous populations are then put in perspective, considering the effect of urbanisation and demographic changes. Further sections examine successively the influence of conflict as a defining element of the resource curse, and the value of mitigation measures at local level. I conclude with a discussion on the marginalised role of health in mainstream resource curse analyses, and with an appeal for considering broader perspectives on causal mechanisms, including indicators of inequalities and social outcomes.

**Table 1:** Core official health agencies operating in a resource curse environment, with their respective attributes pertaining to health services

**Agencies**	**Defining values and operating principles**	**Legitimacy or operational space**
**Governmental health agencies**	social contract, community leadership, laws and regulations	political and administrative mandate over the considered territory
**Non-profit non-governmental organisations **e.g.: local or international NGOs, faith-based organisations, voluntary organisations	e.g.: altruism, solidarity, humanitarian principles	e.g.: humanitarian space
**Corporate oil sector, including transnational corporations**	maximal financial return on investment; corporate social responsibility	operating permit from regulators; social license to operate within 'host' communities

## Results

### Analytical framework for health systems in resource curse environments

The definition of health systems is open to interpretation and, depending on individual points of view or values, it can encompass increasing circles of inclusiveness among activities that define a society [16] (pages 7–8). Moreover, the extent to which health systems contribute to the health of populations is disputed [17]. There is nevertheless a general consensus that health systems are at least one significant element among conditions to achieve better health, aside from a wider range of social and political determinants. Importantly, health systems are also core social institutions with intrinsic values beyond their operational effects [18], trust being an essential cross-cultural value in this respect [19].

The current definition endorsed by WHO [20] draws from considerations by Murray and Frenk [21] who represent health systems as rooted in 'health action'. A health action is defined as 'any set of activities whose primary intent is to improve or maintain health' and a health system encompasses 'the resources, actors and institutions related to the financing, regulation and provision of health action'. Key to these definitions is the notion of *primary intent*, which helps set up the boundaries of a health system among all activities whose effects are to improve health. In the analytical framework put forward in Figure 1, core agencies constitute a tripartite model that encompasses the main categories of health services providers classically present in a resource curse environment: governmental health agencies (GOV) represented by ministries of health and dependent agencies at regional and local level; non-profit non-governmental organisations (NPNGO); and the corporate extractive sector (CES), in this case the oil sector represented essentially by transnational companies and their national subsidiaries. This model assumes an early stage of industrial deployment in a resource curse 'enclave' where mostly *rural areas *would be affected. Later stages of development entail additional complexities due to urbanisation, and will be discussed in a further section. For the sake of simplification, this framework considers only the official providers of health services and it ignores the informal private sector (e.g. private pharmacies), as well as the overlapping category of traditional health practitioners. The latter two categories are popular and probably important in terms of numbers of providers ^b ^[22], but their effectiveness is often very low [23] and their range is difficult to quantify, especially in contexts where regulatory policies are lacking or are not enforced. Excluded also from this model are private, regulated, for-profit health service providers, a category which classically operates in urban areas.

Although the three core categories considered in the framework provide services based on 'western' paradigms of healthcare and operate within the same geographical boundaries, they are clearly different in their underlying values, operating principles and self-defined legitimacy, as illustrated by examples in Table 1.

While government agencies are well defined by national policies and laws, the category of NPNGO providing health services is heterogeneous, and includes faith-based and humanitarian organisations, both further categorised as national or international agencies. Their defining values are generally altruism [24] or solidarity, but many international NPNGO adhere also to the operating humanitarian principles defined by the Red Cross and Red Crescent Movement. Furthermore, they operate in a 'humanitarian space' [25], which can be open to a larger range of actors than international humanitarian organisations. As illustrated in the country examples (Additional file 1 and Appendix 1) and examined in a further section, various types of armed conflicts (ongoing or latent) often characterise resource curse environments, explaining why humanitarian organisations are classically part of this health system, together with other NPNGO and government services. Van Damme *et al*. [26] have shown the functional antagonism that frequently arises between primary health care and 'emergency medical assistance', recognizing that many situations in the developing world have to accommodate a blend between both paradigms. This conjunction of GOV and NPNGO, including emergency humanitarian organisations, is not an uncommon situation in conflict or post-conflict areas, and there is nothing that makes it specific to a resource curse environment. However, a definitely unique feature of healthcare in a resource curse environment is the real or claimed contribution of the CES to health services. In the absence of independent field data, it is impossible to assess accurately whether the CES makes a quantitatively important difference in terms of the share of services provided or beneficiaries attended. However, the proposed analytical framework intends to address qualitative elements as well. This will be illustrated in the next two sections, which focus principally on the CES.

### Role of the corporate sector: corporate social responsibility and social license to operate

It has become popular for the corporate sector to be engaged in a number of health actions covering a range of public health endeavours, such as: supporting global or regional health initiatives, sponsoring biomedical research, sponsoring non-governmental organisations or, more directly, financing local health projects. As an example, the case of Exxon Mobil illustrates the diversity of such contributions through its involvement in malaria control [27]. Obviously, health systems are not value neutral. What defines a health system is much more than the sum of all contributions (financial, material, human) to health services. Values, operating principles, legitimacy and governance are especially important to examine here. These issues will be reviewed respectively through the concepts of: corporate social responsibility, social license to operate and international norms.

Corporate social responsibility (CSR) is a distinct and rather recent operating principle originating from the commercial sector. Definitions of CSR are loose ^c^, resulting in some confusion over its scope [28,29]. CSR is one among several efforts by private companies toward self-regulation of their social standards. A key feature of CSR initiatives is their voluntary character, falling outside imperatives of legal compliance. Watts [29] (p. 9.22–9.23) lists a number of reasons why CSR initiatives are particularly appealing to the oil industry, including a long history of environmental and human right issues that have tarnished the industry's reputation. A market logic is still the underlying principle here [30], but CSR addresses concerns over sustainability in all its dimensions: economic, environmental and social [28]. It is useful to distinguish two health aspects of CSR: public health protection of the company's workforce and protection of the 'host' communities. In practice, CSR achievements by extractive industries are much more impressive for the former than the latter beneficiaries, resulting in hubs of local corporate health services offering the highest standards of care, and typically insulated from surrounding communities ^d ^(Figure 1). This raises important issues of access and equity, and any health system evaluation should specify the exact rules governing access to those insulated health services. Frynas [31] has extensively analysed the motivations, operational effects and developmental linkages of CSR activities in which multinational oil companies claim to be engaged. Motivations seem to be limited to the 'business case for CSR', and include typically: (i) keeping competitive advantage in obtaining territorial concessions; (ii) maintaining a stable and peaceful working environment during critical industrial operations; (iii) improving public relations, often in response to anti-oil protests [32]; and (iv) improving employees' morale. This has typically lead companies to engage in uncoordinated initiatives, with low developmental impacts, short-term scopes, inadequate community consultation processes, and a preference for infrastructure projects over human capacity building. Some oil companies have evolved toward supporting smaller, grass root self-help projects in collaboration with non-governmental organisations or external development agencies. These initiatives are clear operational improvements, but they fail to compensate for resource curse effects on country governance in the health sector. Frynas concludes that 'Perhaps the key constraint on CSR's role in development is the business case, that is, the subservience of any CSR schemes to corporate objectives'.

Related, but distinct from CSR is the concept of 'social license to operate' (SLO), which is the main operational objective of CSR at community level, giving the corporate industry its share of informal legitimacy and additional operational space (Table 1). How the two concepts of CSR and SLO are supposed to apply to community health and interact through their business-oriented logic is best clarified in an illustrative paper from the British Overseas Development Institute:

"Social investments in local health (...), skills and infrastructure improve the capacity to absorb positive spillovers from and enhance linkages with businesses. The concept of absorptive capacity plays an important and positive role in the theory of FDI and development...

At the same time, businesses also have an incentive to make social investments through partnerships over and above the developmental needs of the local people. Such investments will improve local skills, motivation and health of the local workforce, and thus create more efficient labour inputs and higher quality local suppliers on which business become increasingly dependent. Efficient labour inputs and the quality of local suppliers improve business efficiency, while the consent of the local communities provides a 'social license to operate"' [33].

Lee and Bialous [34] advocate for a 'more critical debate within and beyond the public health community on the rapid proliferation of CSR initiatives'. Likewise, the same critical debate should address ethical standards and public health objectives of health initiatives initiated by the corporate sector, whenever a 'social license to operate' is at stake.

A last point to consider here is the nature of health governance regimes under which the corporate sector operates in resource curse environments. By essence CSR entails *self-regulated *norms, and resource curse environments are characterised by poor state governance or deficits in the rule of law. It is therefore important to examine if any international convention would cover norms regulating health systems governance in this context. Article 12 of the International Covenant on Economic, Social and Cultural Rights addresses a number of issues directly relevant to health in resource curse environments. These are specified in General Comment No. 14 issued by the UN Economic and Social Council under 'the right to the highest attainable standard of health' [35], notably: the principle of non-discrimination in accessibility to health facilities, goods and services; the right to healthy natural and workplace environments; and the recognition of adverse health effects due to 'development-related activities that lead to the displacement of indigenous peoples against their will from their traditional territories and environment, denying them their sources of nutrition and breaking their symbiotic relationship with their lands'. These norms legally apply to signatory States parties, and the private sector is not considered under 'Obligations of actors other than States parties'. Regrettably, transnational corporations thus operate within health systems under the same kind of 'governance gap' as described by Gagnon *et al*. [36] for international human rights and humanitarian law. Self-regulation under CSR initiatives can probably compensate for some aspects, but certainly not for the essence of this governance gap.

### Partnerships and contractual relations in the health sector

The three core categories of agencies introduced so far in the analytical framework (Figure 1) entertain naturally a number of contractual or more informal relationships with each other. A number of possible configurations (bipartite or tripartite) can be envisaged.

First, GOV and NPNGO interact classically through informal trust-based relationships or relational contracts. However, multilateral development agencies are currently promoting more binding relationships through contracting-out of health services, as indicated in the case of Southern Sudan. The benefits of this experimental approach are still disputed [37,38]. For primary health care services, system-wide effects are open to question [39].

Second, CES and GOV partnerships in the health sector can be biased by political agendas, at the expense of social achievements. Frynas [31] and Le Billon [40] both give examples pertaining to Angola. Another example of ambiguous partnerships is illustrated by the recent announcement that the current Minister of Health of the Government of Southern Sudan joined the board of advisers of 'Jarch Management Group Ltd.', a US private investment company claiming disputed rights over some oil concessions in the Greater Upper Nile region [41]. The company statement does not mention if the rationale for the partnership with the Minister of Health entails health-oriented CSR projects in the contested area.

Third, the relationship between CES and NPNGO has some underlying complexity. For example, oil companies are unable and unwilling to undertake comprehensive development projects and they are clearly looking for partnerships and joint initiatives to achieve their corporate social responsibilities [42,43]. For extractive industries in general, this strategy has been formalised as a 'tri-sector partnership model of social management' between the government, civil society organisations and the corporate business [33]. From the corporate sector's side, this is described as a 'relatively innovative management technique' for the 'complex social issues relating to FDI in the extractive industries', responding in part to 'the needs of companies to operationalise their corporate social responsibilities at reasonable and sustainable cost' [33]. At international level, a scaled up version of the model ('Tri-Sector Partnering') has been promoted by the World Bank Group as "a management tool that delivers benefits to communities affected by investments and, thus enhances the informal, social 'license to operate' of the investing companies" [44].

With the notable exception of international humanitarian organisations, the 'tri-sector partnership model' promoted by the corporate sector and multilateral development agencies thus draws from the same categories of actors that define the core elements of the formal health sector in a resource curse environment.

### Role of official development assistance (ODA) programmes

Whether implemented under the umbrella of bilateral or multilateral development agencies, the place of ODA-financed health programmes in this context is ambivalent. They dwell upon the core categories of the tripartite health system model illustrated in Figure 1, and borrow similar values to some extent. However, ODA policies represent distinct core values, typically (in the case of bilateral agencies) reflecting the foreign policy and national interests of the country or alliances that they represent [45]. This is what distinguishes ODA programmes from humanitarian assistance, the latter remaining within the remit of soft-power foreign policy [24]. Yet, humanitarian assistance itself can be instrumental to foreign policy, as shown by Middleton and Keefe [46] in the example of Sudan. Furthermore, ODA policies fulfil multisectorial objectives, and thus represent also the commercial interests and defining values of the transnational companies with which governments enter into partnership at the level of higher politics.

### Prospect for evolution

The tripartite health system framework described in previous sections is a dynamic model and it is naturally bound to evolve. Demographic pressure and urbanisation are obvious motors of change for local communities (especially indigenous populations) impacted by the resource curse, either through in site infrastructure developments or, more commonly, through migration toward booming urban areas. To different extents, the four areas examined in this paper are undergoing rapid demographic changes, which result in increased and mostly unregulated urbanisation. This is obvious for Luanda, the capital of Angola, and for Port Harcourt, the capital city of Rivers State, Nigeria. In the Doba basin of southern Chad, the oil extraction area of Komé has doubled its population since 1993 [47]. In Southern Sudan, state capitals like Juba, Wau and Malakal are set for rapid urban growth [48]. Aside from voluntary movements of post-conflict returnees, examples abound to show how oil extractive industries are disruptive of traditional lifestyles and rural communities, and how they constitute a powerful drive toward urbanisation, independently of frequent territorial seizures and forced displacements. The reasons are varied and synergistic, including: environmental degradation; persistent conflicts; loss of agricultural assets and of food security; loss of cultural identity; demographic and social pressure from the in-migration of job seekers [9], and other societal changes related to new job markets ^e ^[13]. Even from a strictly economic perspective, extractive industries can have imbalanced impacts, depending on the geographical level of analysis. In this context, te Velde [33] acknowledges that '...a cost-benefit analysis of an FDI project is likely to lead to different assessments depending on the target group, e.g. national economies versus local communities'.

Harpham and Molyneux [49] have reviewed evidence showing that sub-Saharan Africa is actually the theatre of an 'urban penalty' phenomenon, as far as secular health improvements are considered. Infant mortality rates in particular have risen in small and medium-sized African cities, part of the reason being probably the HIV/AIDS epidemic. Thus, assessments of actual effects of oil extraction projects on 'host' communities should take into account longer-term effects due to urbanisation and they should consider health outcomes and impacts occurring at the actual sites of relocation, particularly when this entails exposure to new social contexts and different determinants of health.

### Violence and conflicts

The association between oil extraction and armed violence is well established, and it is classically considered one of the root causes of the resource curse. First, interstate conflicts are a recurrent theme in the history of oil extraction [29] (p. 9.8–9.10). Second, most of the sub-Saharan countries endowed with substantial oil reserves have been the site of recent or protracted conflicts and violence of some sort. These include civil wars, inter-ethnic conflicts, interstate disputes and military interventions, political repression, human rights abuses [50-52]. Third, oil booms frequently result in the misappropriation of oil revenues by rulers of rentier states, bloating the share of the national budget allotted to military expenditures ^f ^and/or to the weapons industry. Sudan [53] (p. 18) and Chad [54] (p. 10) are classical examples. Fourth, there are complex relationships between transnational oil companies and the security apparatus of their host governments [29] (p. 9.18–9.19), leading in extreme cases to their complicity with security forces in perpetrating human rights abuses [32,55]. Finally, the accumulation of adverse political, economic and social effects brought about by oil extraction at local level can create grievances that lead to armed conflict [15]. A typical example is the ongoing political violence in the Niger Delta region [56].

As a particular form of armed conflict, civil war has been studied extensively, producing a rich and at times inconclusive body of academic literature. Definitions of civil war are not standardised, but they generally entail a specified threshold number of casualties over a time period within a defined context of rebellion [57,58]. The peculiar importance of civil war here is that adverse health effects are considerable and extend well beyond the period of active warfare [57].

Using econometric analyses, Collier and colleagues [59,60] have examined the links between natural resources and civil wars. They claim that there is a direct and highly significant relationship between national dependence upon primary commodity exports (oil in particular) and the risk of internal conflict in low-income countries. In an attempt to explain this relationship, Collier and Hoeffer [61] argue that the *initiation *of rebellions is better predicted by the funding opportunities offered by access to natural resources, than by proxy indicators of social grievances. This notorious theory of 'greed vs. grievance' (more appropriately summarised later by their authors as 'atypical opportunities' vs. grievance) addresses an important development issue. However, the theory has been criticised by independent evaluators for its 'lack of appropriate conceptual and empirical framework' ^g ^[62] and its relevance has been disputed by several scholars [29,63,64]. Ross [12,65,66] provides in-depth reviews of the large body of research available on the links between natural resources and civil wars, explaining why Collier and Hoeffler's findings are actually not robust. Ross's thorough analysis points out to a number of methodological issues (in particular around semantic and parametric definitions) and to a variety of plausible causal mechanisms which have been insufficiently addressed. I would add that the extent to which parameters reflecting grievances have been explored in this body of literature is remarkably poor. For example, in the regression model tested by Collier and Hoeffler [61], none of their proxy measures for grievance relates to social conditions in general, and to health in particular. Using Shell in Nigeria as a case study, Rieth and Zimmer [67] have shown that a transnational company can evolve under the pressure of civil society organisations, toward internalisation of social norms leading to an active role in conflict prevention. Longitudinal observations of this sort suggest useful methodological complements or alternatives to the common cross-sectional parametric approach underpinning the bulk of the 'greed and grievance' literature.

Having reviewed the evidence for armed violence as a leitmotiv in the landscape of oil extraction, the key question is: to what extent does violence contribute to adverse health effects in a resource curse environment? Coupland [68] has clarified the conceptual background and shown that armed violence contributes to health impacts in two ways. The first (and obvious) element is the direct effects of trauma from weapons. The second element is people's insecurity, the latter term being understood in its broad sense encompassing the systemic effects of violence on communities and health services, and quite distinct from national or international security issues. Furthermore, violence as a constitutive element of a resource curse environment justifies the presence of humanitarian actors or other substitutive health organisations, and thus contributes to the perpetuation of a fragmented palliative health system, with indirect effects on the distribution of human resources for health. In their review of the role of health in internal stability and failed states, Lee and McInnes [69] conclude that there is yet no direct evidence to show that 'ill health can contribute to internal instability' or 'whether improved health and better healthcare provision can stabilise states'. It is thus premature to describe the relationship between violence and ill health as a vicious circle in a resource curse context, although lack of access to health services can certainly constitute a major and legitimate source of grievance.

### Health impact assessment and other social impact mitigation measures

There are at least three processes through which adverse health effects of industrial development projects can be mitigated at community level, and which would apply directly to oil extraction. These are: health impact assessments, consultations with local communities, and long term monitoring. A Health Impact Assessment (HIA) is an essential part of the broader Environmental Impact Assessment (EIA) process that is now considered standard practice for project proposals submitted to international development agencies [70]. Lee et al. [71] see HIA as a tool for public health to influence foreign policy. The authors summarise the positive effects of HIA in general by their capacity to (i) raise awareness among decision-makers, (ii) assess the potential impact of specific proposals on populations' health and (iii) improve and optimise the outcome of proposals. From the corporate side, EIA is seen as 'a tool to secure the social license to operate' and a strategy to advance tri-sector partnerships [72]. HIA are less likely to improve the social components of projects (including health issues), compared to strictly environmental issues addressed by EIA [72]. Furthermore, current practices in carrying quantitative HIA suffer from insufficient standardisation and methodological uncertainties about their reliability and validity [73].

If one considers that the 'Chad Cameroon Petroleum Development and Pipeline Project' (CCPDPP: see Appendix 1) is a model of environmental and health impact mitigation in oil extraction (due to the oversight of the World Bank Group), the weight given to public health concerns and to HIA as a guarantee of best practices is at best disappointing. While the preliminary HIA had arguably induced some improvements in specific health outcomes (e.g. malaria, traffic accidents, minor sexually-transmitted infections), broader systemic health issues raised by the international panel of experts appointed by the World Bank were ignored or dismissed by the Consortium of corporate stakeholders [74]. One of the appointed experts asserts that: '...it appeared that in this project decisions were based largely on cost and profit considerations, giving only passing attention to environmental and social aspects, and little or no decision-making power to the affected populations' [74]. Such 'decision making power' is often confounded or misrepresented by the corporate industry as the outcome of 'consultations', another standard practice often included in the CSR package of activities. As pointed out by analysts of the impact of extraction industries on indigenous communities [75], 'consultations are fundamentally flawed as a mechanism to assure that indigenous people rights are fully respected'. The authors point out frequent reasons, including the facts that: (i) companies and governments bias consultation toward obtaining local acceptance of project, (ii) companies and governments fail to disclose critical information to communities about petroleum impacts and (iii) communities are not advised that they are being 'consulted'. In addition, there is a risk that prospects for improvement of health services (typically infrastructure projects) would be used as bargaining power during any consultation process. It should be kept in mind that, ultimately, concerned communities have no veto right upon an industrial project that would impact their territories and threaten their identity, a striking asymmetry of power that is implicitly acknowledged by the World Bank [76] through the existence of a detailed 'involuntary resettlement' policy, however strict are its written safeguards.

Finally, longitudinal monitoring of health outcomes and impacts of projects such as oil extraction should be standard procedures. Taking again the CCPDPP as an alleged model, there is much scope for improvement in practice, with a need for systematic baseline studies and pre-established public health surveillance mechanisms.

## Discussion

The presence of extractive industries in oil-rich areas affects directly the health of local populations. Examples of adverse health outcomes and impacts include: direct effects of environmental degradation, increase in road traffic accidents, acceleration of the spread of HIV and of other sexually transmitted infections. In addition, in oil-dependent countries complex systemic effects interact to determine broader consequences on health, such as: higher rates of child mortality, lower life expectancy, higher malnutrition rates or lower spending levels on health care [15]. Similarly, adverse effects have been described for education [7,13], suggesting that more upstream elements determining the quality of life are at stake in oil-driven development. A health system perspective centred on local communities provides further insights into social determinants of the resource curse, and offers an opportunity to dissect the connections between economic development, poverty and health. Considering the lack of comprehensive analysis currently available to describe health systems in their relationships with resource curse environments, the analytical framework proposed in this article is a first attempt to define important components, linkages and dynamics of such a system. The framework is designed to guide field research as well as stakeholder analyses, and to accommodate both quantitative and qualitative approaches. The three core components (governmental health agencies, non-profit non-governmental organisations and the corporate extractive sector) should be considered with equal importance when determining respective inputs to the system, and when measuring performance indicators which are genuinely relevant for local populations, such as coverage, access and participation. The proposed framework also challenges the current WHO definition of health systems in two aspects. First, the *primary intent *criteria would formally exclude the corporate component (CES) of the core agencies depicted in Figure 1. As reviewed in previous sections, the actions of extractive companies within the health sector (under a CSR agenda) do not have health improvement or maintenance as their primary intent, but instead operational objectives linked to corporate interests, such as the social license to operate. This issue is partly semantic, but it shows the limitations of Murray and Frenk's framework and, by extension, the difficulties to define a health system. Exclusion of the corporate component would also artificially conceal or minimise the contribution of the satellite hubs of healthcare services indicated in the figure, together with the equity and accessibility issues that they raise. Second, the proposed framework (including Table 1) suggests a more comprehensive qualitative approach than the rather vague notion of primary intent. It highlights instead the importance of analysing values, operating principles, legitimacy and operational spaces, as well as the nature of relationships (contractual or informal). In this article this is examined more in depth for the CES, since issues of operational relevance, ethics, governance and regulatory frameworks are more obviously at stake with this component. Similar qualitative analyses could however be carried out for other components of the system such as NPNGO, although the latter are in principle embedded in the health system in a more straightforward and coherent way. The complexity of contractual relationships has also been illustrated in this paper, to show the danger of possible biases resulting in irrelevance, inequity, incoherence or transience of health actions typically driving health systems in resource curse environments. Another danger is that health reforms proposed by development agencies in resource cursed countries (as suggested in the case of Southern Sudan: see Appendix 1) would reinforce the contractual or commercial character of such relationships between actors, at the expense of trust and community values.

Aside from direct health effects of oil extraction, Figure 1 puts the analytical framework for health systems in relation with currently identified (economic and political) elements of the resource curse phenomenon. This does not necessarily imply established causal mechanisms, but it simply suggests a number of plausible links by which resource curse findings could affect the structure, function or perpetuation of a peculiar health system. Obviously, more research needs to be done on these links. Adverse social effects (and health effects in particular) could ultimately appear to represent more upstream elements among resource curse mechanisms.

I argue that health (as a social and community value) has been *marginalised and instrumentalised*, not only in the concrete contexts in which extractive industries operate, but also in mainstream development discourses proposing remediation to resource curse situations. Marginalisation of health has been exposed throughout this paper: (i) by the dominance of econometric parameters to define the resource curse, (ii) by the lack of a longer-term analysis taking into account the health consequences of urbanisation and (iii) by the poor weight that HIA and other health mitigation measures carry in the face of economic interests. Instrumentalisation of health appears in: (i) the nature of the operational concept of 'license to operate', (ii) the corporate perspective on HIA as an instrument to secure licenses to operate and (iii) the type of contractual partnerships promoted by multilateral development agencies, including tri-sector partnerships.

One might wonder why, with few exceptions, health and other social parameters of well-being have not received more attention in the resource curse literature until recently. Reasons might be historical or methodological, but also ideological. For example, the possibility of health as an explanatory variable is conspicuously absent from a recent authoritative textbook [14] on 'Escaping the resource curse' ^h ^co-authored by Jeffrey Sachs. This is still more troubling as one remembers that the same author has prominently been leading research and political agendas valuing health as a major determinant of economic growth [77].

As mentioned in the introduction, initial econometric findings on the resource curse are reductionist in their scope (countries vs. affected communities) and in their perspective (economic and political factors vs. social outcomes). The theoretical foundations of this reductionist perspective obviously reflect mainstream development theories and macro-economic policies supported by international financial institutions. Perpetuating an exclusively economic and political research agenda would carry the risk to see ideologically biased solutions prescribed prematurely, while ignoring other important and neglected dimensions of the resource curse phenomenon. Remediation measures to armed conflict proposed by some analysts of the resource curse are indeed biased toward macro-economic interventions. Bannon and Collier [60] (p. 8–11) offer an illustrative example in this respect. Essentially, such orthodox remediation theories to the resource curse are convenient constructions around a 'dominant paradigm' [78] of development. This paradigm promotes accelerated growth and opening to global markets as essential pillars of development and poverty alleviation, and justifies the large scale extraction of natural resources as an economic primacy and an inescapable necessity. Unwittingly, the current and incomplete corpus of 'resource curse' findings allows the conceptualisation of what is simply an exemplary failure of dominant development paradigms, sanctioning the issuance of self-serving prescriptions, and avoiding the direct questioning of their relevance to genuine social outcomes. Commenting about such prescriptions, Lahiri-Dutt [64] notes that: 'they do not question the legitimacy of the system of resource governance to raise uneasy issues such as community rights over the local resources'. For destitute villagers in the Niger delta, the 'real GDP growth per capita' has absolutely no relevance. What counts is their quality of life and how to improve it. Chambers [79] has provided a remarkable analysis of the multiple and complex dimensions of poverty or well-being, and related perceptions. The choice of indicators to define such complex realities is also revealing of methodological biases in resource curse theories. The GDP is a measure that is oblivious of gross inequalities and, among other shortcomings, it includes the product of illicit or socially adverse activities. Some economists have therefore proposed to substitute the GDP by a more accurate 'Genuine Progress Indicator' (GPI) to measure economic performance as a better reflection of well-being [80]. Furthermore there are more encompassing indicators of development, such as the Human Development Index (HDI) which was launched as early as 1990 [78] (p. 205–206), and which compounds measurements of life expectancy, level of education and income [3]. Surprisingly, despite increasing recognition by policy makers of the multiple dimensions of deprivation, purely 'economic' measures of poverty still have a higher status among key development indicators [81]. Beyond popular but myopic discourses on 'poverty reduction', there is thus a need for new conceptual approaches to the resource curse, and for a definition that would focus on social outcomes, instead of economic determinants. Whatever indicator is chosen, it should also be sensitive to inequalities, both within oil-producing countries and within impacted communities. Monitoring health indicators is especially important to consider in this respect.

At this point, mention should be made of two countries that are classically singled out as evidence that the resource curse is escapable, provided that proper governance, transparency and economic policies are in place. Norway is an oil-producing country, which has some of the highest-ranking development indicators in the world (Additional file 1). It is considered a model of oil revenue management, including establishment of an oil fund for the sustainable financing of retirement and health insurances [10]. The Norwegian government actually controls most of oil revenues through taxes and fees, oil wealth being a common-property resource by law in this country [7]. While oil production has resulted in recent economic growth, Norway, like other Scandinavian countries, had strong social and redistributive public policies in place well before oil exploitation could produce any economic effect [82]. It would thus be an oversimplification to attribute social welfare in Norway to the mere effect of oil-driven growth.

Botswana, the 'fastest-growing economy in the world' [60] is a more complex case. Let us put aside the fact that diamonds deposits (discovered in 1967), instead of oil, is the natural resource asset here. Arguably, good governance, maintenance of traditional and political institutions [8] and transparency over diamond revenues [83] explain the good economic performance of Botswana. Nonetheless, and despite a high public spending on health and education [7], life expectancy at birth remains low (48.1 years in 2005). This fact is generally attributed to the AIDS epidemic, which has dramatically affected the country. Such discrepancy is troubling and suggests that economic wealth in Botswana has not translated in better control over the social and political determinants of the spread of HIV/AIDS [84]. On closer look ^i^, Botswana does not perform that well in terms of equity [85] and genuine democratic process [86]. Taylor and Mokhawa [87] see the forced destitution of the San Bushmen from their ancestral homes as a significant form of conflict, although this has not reached the magnitude of a civil war which would signal the presence of another defining element of the resource curse. In addition, the 'GDP per capita rank minus HDI rank', (an indicator of performance in translating the society's wealth into social development) is currently the lowest for Botswana (minus 70), among all countries considered in the latest Human Development Report [3]. The cases of Norway and Botswana are thus important to illustrate once more how purely economic indicators are profoundly distorting the exact nature and magnitude of the resource curse. A more accurate definition should include a range of indicators of equity and social well-being, such as health outcomes and impacts, and it should be open to a full and unbiased set of plausible causal relationships.

## Conclusion

As we are probably approaching the peak of world oil production [88], it is likely that an increased frenzy of oil exploration and exploitation will plague more rural communities in non-industrialised countries and create more resource curse environments rather than Norwegian-style paradises. Sustainability of oil wealth through incremental extraction (in proportion to local or national needs) is not on the agenda of development organisations. This is another sign that oil-driven 'development' is not geared to benefit local communities, but instead to sustain the viability of global markets and the acute needs of industrialised countries. Some industrialists [89] and some members of the World Bank [11] (p. 20, footnote 99) alike dispute the 'resource curse' concept, preferring 'governance curse' as a qualifier. As far as oil extraction industries represent an epitome of mainstream development policies and of the mantra of rapid economic growth, impacted communities would rather see oil as a 'development curse'.

## Appendix 1: country context analysis

### Niger Delta region (Nigeria): fragmented health services amidst rebellion

Nigeria, the world's eighth-largest oil exporter, has a three-tiered federal government system with 36 states divided into 774 local government councils (LGC). The country gained independence in 1960 and started oil exploitation in 1958, essentially in the Niger Delta region, where four states (Akwa Ibom, Bayelsa, Delta and Rivers) account for most of the national production. In spite of such wealth, this is a region where the GNP per capita and educational levels remain below national average, and 70 percent of its 20 million people are living below the poverty line ^j ^[63,90]. The environmental impact of oil production has been disastrous, due to frequent oil spills and to the common practice of gas flaring [43,90-92]. Deforestation and quarrying activities to meet the needs of rapid and uncontrolled urbanisation [93] add to the burden of environmental degradation. Livelihoods have traditionally relied upon fishing and farming. These vital resources are now severely compromised by the effects of water pollution, degradation of arable soils and land seizure [90]. Conflict and violence have been persistent since the 1990's, due to multiple and intricate factors [22,63]. This has created a climate of rebellion and insecurity, culminating recently with the frequent kidnapping of foreign oil workers, a threat to the sustainability of industrial investments [94]. Political repression [56] and massacres of rebellious communities by official authorities are well documented, for instance the Umuechem massacre of 1990 [43]. The Niger Delta region is more affected by HIV infection than any other region or zone in the country, due to the combined effects of poverty, urbanisation, unemployment, and migration of foreign or national labourers [95].

As shown by Human Rights Watch [56] in the Rivers State, corruption is overwhelming in most of the LGCs surveyed. Social services (health and education) and facilities run by LGC authorities in the region have collapsed [93]. Itinerant drug sellers and traditional healers are frequently used as substitutes for public healthcare [22]. Throughout the country, primary health care services are under the responsibility of LGCs, but in practice, they are delivered in large part by non-state providers, in particular by faith-based organisations affiliated with the Christian Health Association of Nigeria [96]. Due to increasing levels of violence, international humanitarian organisations such as Médecins Sans Frontières [97] maintain an operational presence, mostly in Port Harcourt, the capital of the Rivers State. The role of transnational oil corporations and their subsidiary companies in contributing to community development programmes in general ^k ^[29], and in the provision of health services in particular is unclear and controversial. Shell for instance is upbeat about its involvement in immunisation campaigns, HIV/AIDS awareness campaigns, and support to health facilities and services [42,98]. However, independent evaluations [22,32,43] as well as anecdotal press reports show that the reality on the field is far from matching such claims of social achievements. As a symbol of two diverging worlds, the contrast is blatant between the quality of health care offered at the Shell hospital inside of the company compound in Port Harcourt, and the derelict state of public health facilities in the region [99].

### Angola: offshore wealth and foreign aid

With huge reserves of mostly offshore oil and a population of 12.4 million, Angola is one of the richest countries in Africa. Yet, it ranks poorly in terms of social development and it has the highest level of inequality among oil and gas producers. At least one third of Angola's population resides in shantytowns [100] with very limited access to clean water [54]. The year 2002 marked the end of 27 years of civil war between UNITA rebels funded by the diamond trade and the governing MPLA financed by oil exports [50,100]. Oil revenues have sustained a war effort against the armed separatist rebellion, which claims sovereignty over the Cabinda enclave, a territory including 60 percent of Angola's oil assets [40]. In August 2006, a peace agreement was signed between the government and rebel forces in Cabinda.

The ruling elite of Angola is opaque and largely unaccountable about national oil revenues amounting to several billion US$ per year. The country is still very much dependent on foreign aid for the vital sectors of health, food relief and emergency assistance. The transition from humanitarian relief to reconstruction has been slow, partly due to obstructions to the work of aid agencies by the Angolan government. The role of oil companies in the social sector is troubling, and Le Billon [40] could comment that: "As for financial support for social, economic and humanitarian projects, oil companies have become one of the main sources of private funding. There is some concern, however, regarding the legitimacy and the political nature of some donations, such as those benefiting 'well-connected NGOs'...".

Compared to the Niger delta, Angola thus offers a more straightforward example of the resource curse, devoid of major local and environmental dimensions, while the country at large is still relying heavily on foreign aid and relief agencies for the provision of basic services. For example, excluding landmines action, Angola has appealed for a total of US$ 17,468,992 in foreign emergency assistance in 2007 [101].

### Southern Chad: a World Bank experiment under scrutiny

With the assistance of the World Bank Group (WBG), oil fields have been exploited since 2004 in the Doba basin of southern Chad, through an impressive and elaborate public-private partnership between a consortium of transnational companies, and the governments of Chad and Cameroon. The project includes the development of three oil fields in southern Chad, a 1000-km underground pipeline running through Cameroon, an offshore export terminal, and the construction or rehabilitation of a large number of ancillary infrastructures alongside [11]. The 'Chad Cameroon Petroleum Development and Pipeline Project' (CCPDPP) is a major investment, and an endeavour by the WBG and partners to set up a model of economic, political and social achievements in poverty reduction through oil exports. While unrest and conflict have not specifically reached this southern region of Chad, civil society organisations have expressed strong reservations about the merits of the project, with regard to the endemic climate of corruption, poor governance and political repression [9,102]. Accordingly, the WBG has put monitoring processes in place and, to some extent, it has held to its strong commitment to set a precedent in social and environmental standards. After more than three years however, social achievements of the project at community level are disappointing, despite claims by the WBG that large public constructions are well underway in the cities of Doba and Bébédja [103]. More importantly, the political and economic safeguards that the WBG had required to avoid a resource curse are being jeopardised, and expectations of improved governance from national authorities are not met ^l ^[9,102,104]. One important adverse element stressed by external analysts (see for instance [11]) is that, while oil production capacity has been established ahead of schedule, institutional capacity-building projects have badly lagged behind.

The health sector is poorly developed in Chad, due to a lack of qualified medical staff, a lack of management skills at all levels, and a concentration of health services in urban areas [105]. The question therefore is whether the CCPDPP will help improve health outcomes in general, and a deficient health system in particular. As noted by Gary and Karl [102], the Chadian revenue management law of 1998 is 'vague regarding priority sector and regional spending', lacking any '...directive about whether money may be spent, for example, on primary health clinics in rural areas or state of the art hospitals in the capital'. In 2005, this law which was designed to offset resource curse effects was unilaterally abrogated, and renegotiated with the World Bank under more favorable terms for discretionary use of oil revenues by the Chadian government [106]. There are anyway no signs that the existing public health infrastructure has been improved in the project area [11], despite initial warnings by a panel of experts who observed a 'shocking disparity in affluence [between the consortium's health facilities and local hospitals], calling into question the ethical values inherent in this project' [74]. One of the alleged strengths of the project is a community health outreach programme addressing in priority HIV/AIDS, sexually transmitted infections, and malaria [107]. The focus on the former two conditions is critical since the CCPDPP entails an influx of job-seekers and truck drivers, most of them single or unaccompanied men, while up to 50% of prostitutes in some areas around the pipeline are infected with HIV [13,74,108]. The malaria-control program and the HIV/AIDS prevention campaign are both run by local non-governmental organisations [107]. The independent International Advisory Group [109] (7^th ^and 9^th ^statutory missions) has repeatedly noted the absence of baseline studies and delays in setting up monitoring mechanisms, in particular with respect to HIV/AIDS prevalence in the project areas. Health impacts of the project will therefore be difficult to determine.

### Southern Sudan: health reforms throughout a fragile peace process

A glance at a concession map [110] reveals outright how much the geographical and human landscape of southern Sudan is and will be affected by oil extraction and by the presence of transnational companies. After a devastating conflict between the north and the south spanning about 50 years, a Comprehensive Peace Agreement (CPA) was finally signed on January 2005 between the Government of Sudan (GOS) and the Sudan People's Liberation Movement (SPLM). This peace settlement is fragile, due to ongoing tensions between political factions; disputes over the final territorial borders between the Government of National Unity (GNU) and the recently established Government of Southern Sudan (GOSS); and the uncertain fate and livelihoods of returnees, most of them compelled to settle in fast expanding urban areas [111]. Access to oil fields has been one of the major issues in the protracted north-south conflict [54,112], resulting in loss of livelihoods, forced displacements, massive violations of human rights, and ultimately to dramatic health impacts and to the destruction of many rural communities [113]. Evidence for the complicity of some transnational companies with helping the GOS in repeated attacks on civilian populations is overwhelming [55,53] (part 2). The most publicised case involved Talisman Energy, the largest western company involved in Sudan at the time, which ultimately withdrew from its oil operations in the country in 2003, in response to shareholders' concerns over human rights abuses. During the last years of the north-south conflict (2000–2004) and under implicit or explicit political agendas [46,114], the international community spent huge amounts of money in humanitarian aid, essentially as food distributions. The direct monetary contribution to health through this process barely exceeded 5% of the total. The situation of the health sector has been described by the newly established Minister of Health as "dismal, with a system that is severely fragmented across multiple actors and numerous vertical programs, largely unregulated, inefficient, under-funded, with a derelict infrastructure and an impoverished and internally distorted work-force"^m ^[115]. As an illustration of the cumulative impacts of the conflict, a recent Sudan Household Survey showed that Southern Sudan has one of the highest Maternal Mortality Rates in the world (2030 per 100,000 births). The World Bank has been housing a Multi Donor Trust Fund to which the Ministry of Health of the Government of Southern Sudan [115] has submitted a three-years proposal for a health sector development programme. The programme is based on a two-track strategy covering rapid and long-term interventions respectively. The fast track relies on contracting firms or NGOs to implement a pilot project including: the management of large hospital services, the reform of the existing civil health services, and the expansion of basic health services in underserved areas. Given that the long-term health policy of the World Bank for Sudan in general is definitely directed toward the introduction of cost recovery and the privatisation of the health sector [116] (p. 122), it is likely that the GOSS will be compelled to follow the same footsteps. Meanwhile, during its first Health Assembly in June 2007, the GOSS has adopted resolutions towards a decentralised health care system [117]. The exact contribution of the oil industry in the rehabilitation of the public health sector, either in kind or as a contribution to health financing is unknown, although several referral health facilities of South Sudan are known to have been established or rehabilitated by private oil companies. Talisman Energy was one of them [53] (Part 2, p. 26–29). Lessons from the environmental tragedy in the Niger delta do not seem to have been learned in Southern Sudan. For example, recent reports indicate forceful evictions and ongoing environmental degradation by oil companies in the Sudd Wetlands, the world largest swamplands of the world. Cattle livelihoods are being lost. Soils and drinking water are contaminated by saline water injected to maintain pressure of the oil reservoirs, and by the dumping of industrial waste in swamp areas liable to flooding [118]. One resident was quoted saying: "If the government ignores us we will go Nigeria-style [struggle]".

## Endnotes

a. This is at odd with more recent aggregate regional data produced by Sachs [14] (Chapter 7), but methodologies are crude in both cases, reflecting the common use of macro-economic indicators as analytical variables.

b. See: de Jong [22] for the concrete example of Bayelsa State in the Niger Delta region.

c. For reviews of the historical context of CSR and of the variety of definitions, see Harrison [28] and Watts [29].

d. See examples mentioned in Appendix 1, sections on the Niger Delta and southern Chad.

e. For an analysis of oil-related societal changes, and rural-to-urban migration in particular, see Karl [13].

f. This increase in military expenditure is seen in both absolute and relative terms, as mentioned by Ross [15] (p. 15). See also Karl [13] (p. 22).

g. Acemoglu D, quoted in Banerjee [62].

h. In the book [14], health and environment issues are mentioned in a short section (p. 109–110), and exclusively under the angle of legal regimes and litigation.

i. The following references and considerations about Botswana were kindly suggested to me by one of the reviewers.

j. Niger Delta Development Commission (NDDC), 2004: quoted by Idemudia and Ite [63]; see also Aaron [90] for an overview of quantitative data on regional development in the Niger Delta.

k. For an historical overview, see 'Capital and community: a case study' in Watts [29].

l. For a comprehensive analysis of the CCPDPP, see Gary and Karl [102]. For an updated and independent field evaluation, see Koczy and Kofler [104]. For an overall analysis of the role of the WBG, see Pegg [9].

m. Letter of Sector Development Policy by Dr. Theophilus Ochang Lotti, Minister of Health. Annex 12, In: Government of Southern Sudan [115].

## Abbreviations

CCPDPP: Chad Cameroon Petroleum Development and Pipeline Project; CES: Corporate Extractive Sector; CPA: Comprehensive Peace Agreement (Sudan); CSR: Corporate Social Responsibility; EIA: Environmental Impact Assessment; FDI: Foreign Direct Investment; GDP: Gross Domestic Product; GNU: Government of National Unity (Sudan); GOS: Government of Sudan; GOSS: Government of Southern Sudan; GPI: Genuine Progress Indicator; GOV: Governmental health agencies; HDI: Human Development Index; HIA: Health Impact Assessment; LGC: Local Government Council (Nigeria); MPLA: Movimento Popular de Libertação de Angola; NPNGO: Non-Profit Non-Governmental Organisations; ODA: Official Development Assistance; SLO: Social License to Operate; SPLM: Sudan People's Liberation Movement; SSA: Sub-Saharan Africa; UNITA: União Nacional para a Independêncao Total de Angola; WBG: World Bank Group.

## Competing interests

The author declares that he has no competing interests.

## Supplementary Material

Additional file 1 Table 2. Some key features about selected oil-producing countries. Click here for file
