# Supplementary material for: Oil for health in sub-Saharan Africa: health systems in a 'resource curse' environment
Source: Global Health. 2008 Oct 21;4:10. doi: 10.1186/1744-8603-4-10 (PMC2596095; doi:10.1186/1744-8603-4-10)
Supplement: Additional file 1 — Table 2. Some key features about selected oil-producing countries. [file 1744-8603-4-10-S1.doc]

**Table 2: Some key features about selected oil-producing countries**

|  | **Niger Delta Region (Nigeria)** | **Southern Chad** | **Angola** | **Southern Sudan** | Norway |
| --- | --- | --- | --- | --- | --- |
| Human development index (HDI) year 2005 | 0.564^a^ | NA | 0.446^b^ | NA | 0.968^b^ |
| Life expectancy at birth | 43 years (UNDP estimate for 2006)^a^ | NA | 41.7 years (2005)^b^ | 42 years (2001)^c^ | 79.8 years (2005)^b^ |
| Under five mortality (per 1,000) | 103 (2003)^a^ | NA | 260 (2003)^d^ | 250 (2001)^c^ | 4 (2000)^e^ |
| % population living in extreme poverty | 70% (UNDP estimate)^a^; 54.4% (government estimate 2004) | NA | NA | >90% (2003)^c^ | Generic poverty index not applicable |
| Nature of conflict and instability | Local, ongoing political violence | Currently stable in oil extraction area | Post-conflict, returnees. Separatist movement in the Cabinda enclave | Immediate post-conflict, unstable | Not applicable |
| Independence year | 1960 | 1960 | 1975 | 1956 | 1814 |
| Other relevant historical landmarks |  |  | end of civil war: April 2002 | Comprehensive Peace Agreement (CPA): 2005 |  |
| Year of beginning of oil production | 1958^f^ | 2004^g^ | 1956 ^h^ | 1999^i^ | 1971 ^j^ |
| Oil exports dependence (% GDP) | 52% (2007)^k^ | 33.4% (2004)^l^ | 54% (2004)^l^ | NA | 23.1% (2006)^m^ |
| Exploitation sites | onshore and offshore | onshore | offshore | onshore | offshore |
| Stated revenue allocation to oil-producing host communities | 13%^n^ | 5%^o^ | Not applicable | 2%^p^ | Not applicable |

NA: data for the considered indicator are not available at sub-national level.

Sources: ^a^UNDP Nigeria [1]; ^b^UNDP [2]; ^c^UNICEF [3]; ^d^WHO [4]; ^e^UNICEF [5]; ^f^Aaron KK [6]; ^g-h^Wurthmann G [7]; ^i^ECOS [8]; ^j^Government of Norway [9]; ^k^World Bank [10]; ^l^OECD [11]; ^m^Statistics Norway [12]; ^n^Human Rights Watch Report [13]; ^o^World Bank [14]; ^p^USAID [15]: figure taken from the example of the Ngok-Dinka chiefdoms in the Abiey territory.

**References**

1. UNDP Nigeria: **Niger Delta human development report**; 2006 [<http://www.ng.undp.org/reports/nigeria_hdr_report.pdf>]. Accessed 11 October 2008
2. UNDP: **Human Development Report 2007/2008.** [[http://hdr.undp.org](http://hdr.undp.org/)]. Accessed 11 October 2008
3. UNICEF: **Towards a baseline: best estimates of social indicators for Southern Sudan***.* New Sudan Centre for Statistics and Evaluation, in association with UNICEF. NSCSE series paper 1/2004. [<http://www.reliefweb.int/library/documents/2004/splm-sud-31may.pdf>]. Accessed 11 October 2008
4. WHO: *WHO Regional Offices Statistics* 2007 [<http://www.who.int/healthinfo/statistics/regions/en/index.html>] Accessed 11 October 2008. Geneva: World Health Organization.
5. UNICEF: **Infant and under five mortality** 2001 [<http://www.unicef.org/specialsession/about/sgreport-pdf/01_InfantAndUnder-FiveMortality_D7341Insert_English.pdf>]. Accessed 11 October 2008
6. Aaron KK: **Perspective: big oil, rural poverty, and environmental degradation in the Niger Delta region of Nigeria.** *J Agric Saf Health* 2005, **11(2):**127-134.
7. Wurthmann G: **Ways of using the African oil boom for sustainable development***.* Economic Research Working Paper No. 84 [<http://www.afdb.org/pls/portal/docs/PAGE/ADB_ADMIN_PG/DOCUMENTS/ECONOMICSANDRESEARCH/ERWP_84_0.PDF>] Accessed 11 October 2008. African Development Bank; March 2006.
8. ECOS (European Coalition on oil in Sudan): **Sudan’s oil industry: facts and analysis, April 2008.** [[http://www.ecosonline.org](http://www.ecosonline.org/)]. Accessed 11 October 2008
9. Government of Norway, Ministry of Petroleum and Energy: **Chapter 1: Norwegian oil history in brief.** In *Fact sheet: Norwegian petroleum activity*; 2004. [<http://www.regjeringen.no/en/dep/oed/Documents-and-publications/Reports/2004/Fact-Sheet-2004-Norwegian-Petroleum-Activity-.html?id=419441>]. Accessed 11 October 2008
10. World Bank: **Country brief: Nigeria** 2007 [<http://web.worldbank.org/WBSITE/EXTERNAL/COUNTRIES/AFRICAEXT/NIGERIAEXTN/0,,menuPK:368906~pagePK:141132~piPK:141107~theSitePK:368896,00.html>]. Accessed 11 October 2008
11. OECD: **African Economic Outlook 2005/2006.** Country studies: Angola. [<http://www.oecd.org/dataoecd/37/35/36734978.pdf>] and Chad [<http://www.oecd.org/dataoecd/37/22/36735933.pdf>]. Accessed 11 October 2008
12. Statistics Norway: **External economy** 2006 [<http://www.ssb.no/english/subjects/09/ur_okonomi_en/>]. Accessed 11 October 2008
13. Human Rights Watch Report: **Chop Fine. The human rights impact of local government corruption and mismanagement in Rivers State, Nigeria.** 2007 (January), **Vol.** **19**, No. 2(A). [<http://hrw.org/reports/2007/nigeria0107/>]. Accessed 11 October 2008
14. World Bank: **A rekindling of hope in the oil producing region**. World Bank, Washington, D.C; 2007 [<http://web.worldbank.org/WBSITE/EXTERNAL/COUNTRIES/AFRICAEXT/>]. Accessed 23 December 2007
15. USAID: **Sudan: strategy statement 2006-08**; 2007. [<http://www.usaid.gov/locations/sub-saharan_africa/countries/sudan/docs/sudan_strategy.pdf>]. Accessed 11 October 2008
